# Zika virus modulates arthropod histone methylation for its survival in mosquito cells

**DOI:** 10.1371/journal.pone.0319290

**Published:** 2025-02-13

**Authors:** Telvin Harrell, Swarnendu Basak, Hameeda Sultana, Girish Neelakanta

**Affiliations:** 1 Department of Biological Sciences, Old Dominion University, Norfolk, VA, United States of America; 2 Department of Biomedical and Diagnostic Sciences, College of Veterinary Medicine, University of Tennessee, Knoxville, TN, United States of America; Instituto Nacional de Salud Pública: Instituto Nacional de Salud Publica, MEXICO

## Abstract

Zika virus (ZIKV) is a mosquito-borne human pathogen that causes mild febrile illness in adults and severe neurological complications and microcephaly in newborns. Studies have reported that ZIKV modulates methylation of human and viral RNA critical for its replication in vertebrate cells. In this study, we show that ZIKV modulates mosquito S-adenosyl methionine (SAMe)-synthase, an enzyme involved in the production of SAMe (methyl donor), and histone methylation for its survival in mosquito cells. Reverse transcription quantitative PCR followed by immunoblotting analysis showed increased amounts of SAMe synthase at both RNA and protein levels, respectively, in C6/36 mosquito cells infected with ZIKV at day 1 post infection (p.i.). Increased levels of SAMe was noted upon ZIKV infection at day 1 p.i in mosquito cells. In addition, increased EZH2 histone methyl transferase-like gene transcripts and methylated histone (H3K27me3) levels were evident in mosquito cells upon ZIKV infection. Exogenous addition of SAMe to mosquito cells showed increased ZIKV loads and EZH2 histone methyl transferase-like gene transcript levels. Furthermore, treatment of mosquito cells with EZH2 inhibitor resulted in reduced histone methylation and ZIKV loads. Collectively, our study provides novel information in understanding the importance of SAMe and histone methylation in the survival of ZIKV in its arthropod vector.

## Introduction

Zika virus (ZIKV) is a positive-sense single stranded RNA Flavivirus transmitted to humans mainly by infected mosquito bites [1]. Intrauterine, intrapartum and sexual transmission of ZIKV have also been extensively reported [1–3]. In addition, a study also reported ZIKV transmission via blood transfusion [4]. ZIKV was first isolated in 1947 from the blood of rhesus monkey in Zika forest of Uganda in Africa [5]. Human infections with ZIKV was first detected in 1952 with the presence of neutralizing antibodies against the virus [6]. ZIKV infection in humans is normally asymptomatic, but in some could lead to fever, maculopapular rash, myalgia, headache and conjunctivitis [1–3]. In severe cases, microcephaly, birth defects, meningoencephalitis, thrombocytopenia, myelitis and Guillain-Barré syndrome (GBS) have been reported in association with ZIKV infection [1–3]. Only 14 human cases were reported from 1947–2006 [7–9]. However, a dramatic increase in the ZIKV infections was noted since 2007 [3,10–14]. In the Americas, ZIKV infections were first noted as an outbreak in Brazil in 2015 [15–17]. Since then ZIKV infections were reported from several parts of South America, Central America, Caribbean, Mexico and in the United States [16,17]. In 2016, out of total 5, 168 ZIKV cases reported from United States and District of Columbia, 224 cases were presumed to be due to the result of local mosquito-borne transmission [18].

Several *Aedes* species including *A*. *aegypti*, *A*. *albopictus*, *A*. *africanus*, *A*. *luteocephalus*, *A*. *furcifer* and *A*. *vittatus* are most likely the enzootic vectors of ZIKV and other flaviviruses [19,20]. In particular, *A*. *aegypti* has been linked to most of the human ZIKV cases [19]. ZIKV has been identified in more than 25 mosquito species including *A*. *albopictus* [19,21]. Grard et al., have speculated that the increased abundance of *A*. *albopictus* in comparison to *A*. *aegypti* in certain regions could result for the earlier species to serve as a potential vector for ZIKV [22]. Conceptually, ZIKV infection is not overtly detrimental, but persistent for the life of the mosquito; typically 2–4 weeks in nature [23]. Often infection is acquired via ingestion of an infected blood meal, initiating replication within the mosquito midgut. As the virus replicates to higher titers, the virus is disseminated throughout the mosquito body, detectible in the farthest most points including the legs and wing tips. The virus spreads to the salivary glands, where it can be successfully spread to a new host via saliva during subsequent blood meals [24]. The time from ingestion of an infected meal to viral transmission is termed the extrinsic incubation period (EIP) [25]. Many of the mechanisms that occur during the EIP are unclear, complex, and highly influenced by environmental, host and viral factors [26]. General alterations that occur during EIP, have been illuminated by collectively studying flaviviruses include changes in RNAi pathways [23,27], antagonization of antiviral pathways (Toll and JAK/STAT pathways) [28], and alterations in epigenetic patterns to better suit the virus in virus-host interactions [29,30].

Some of the most studied epigenetic modifications includes but not limited to DNA and RNA methylation, histone modifications and the effect on nucleosome location [29,30]. These modifications result in chromatin remodeling and transcriptional regulation important in virus-host interactions [29,30]. A study reported that ZIKV infection affects viral and human RNAs by altering the topology and function of N^6^-adenosine methylation (m^6^A), a modification affecting RNA structure and function [31]. Methylome profiling revealed that ZIKV infection alters m^6^A location in mRNA, methylation motifs and target genes resulting in the changes that affect the host cellular functions [31]. Dengue virus (DENV), another member of the flavivirus, capsid protein has been reported to bind core histones and inhibit the nucleosome formation in human liver cells [32]. Also, vaccinia virus K7 protein has been shown to promote histone methylation during infection [33]. Histone modifications and chromatin remodeling are also common epigenetic modifications during bacterial infections [34].

Eukaryotic nuclear DNA is associated with core histones H3, H4, H2A and H2B [35]. Most commonly, histones are post-translationally modified by acetylation on lysine residue, methylation on lysine and arginine residues, phosphorylation on serine and threonine residues and ubiquitylation or sumoylation on lysine residues [36]. These modifications contribute to the control of gene expression either by influencing chromatin organization or signaling to other molecules [36]. A study has mapped the distribution and levels of two post-translational histone modifications, histone 3 lysine 27 acetylation (H3K27ac) and histone 3 lysine 27 methylation (H3K27me3) in *Anopheles* mosquitoes [37]. It was noted that the profiles of H3K27ac and H3K27me3 were mutually exclusive and were associated with high and low levels of transcription, respectively [37]. Histone methyl transferases are known to catalyze the addition of methyl group from S-adenosylmethionine (SAMe), a common methyl donor, to histones [38]. Flaviviruses, including ZIKV, encode a methyltransferase necessary for capping viral mRNA, protein synthesis, and viral replication [39]. Although the viral encoded methyltransferase utilizes SAMe, ZIKV does not have the ability to synthesize it, depending on a host cell for its production [40]. While much is known about the importance of histone modifications in virus-vertebrate host interactions, very little is known on its importance in virus-vector interactions. In this study, using *A*. *albopictus* mosquito cells, we report that ZIKV induces expression of S-adenosyl methionine synthase and EZH2 histone methyl transferase-like gene that subsequently enhance SAMe levels leading to increased H3K27me3 levels important for the virus survival in the mosquito cells.

## Results

### ZIKV induces S-adenosyl methionine (SAMe) synthase expression in *A*. *albopictus* C6/36 cell line

**Fig 1:**
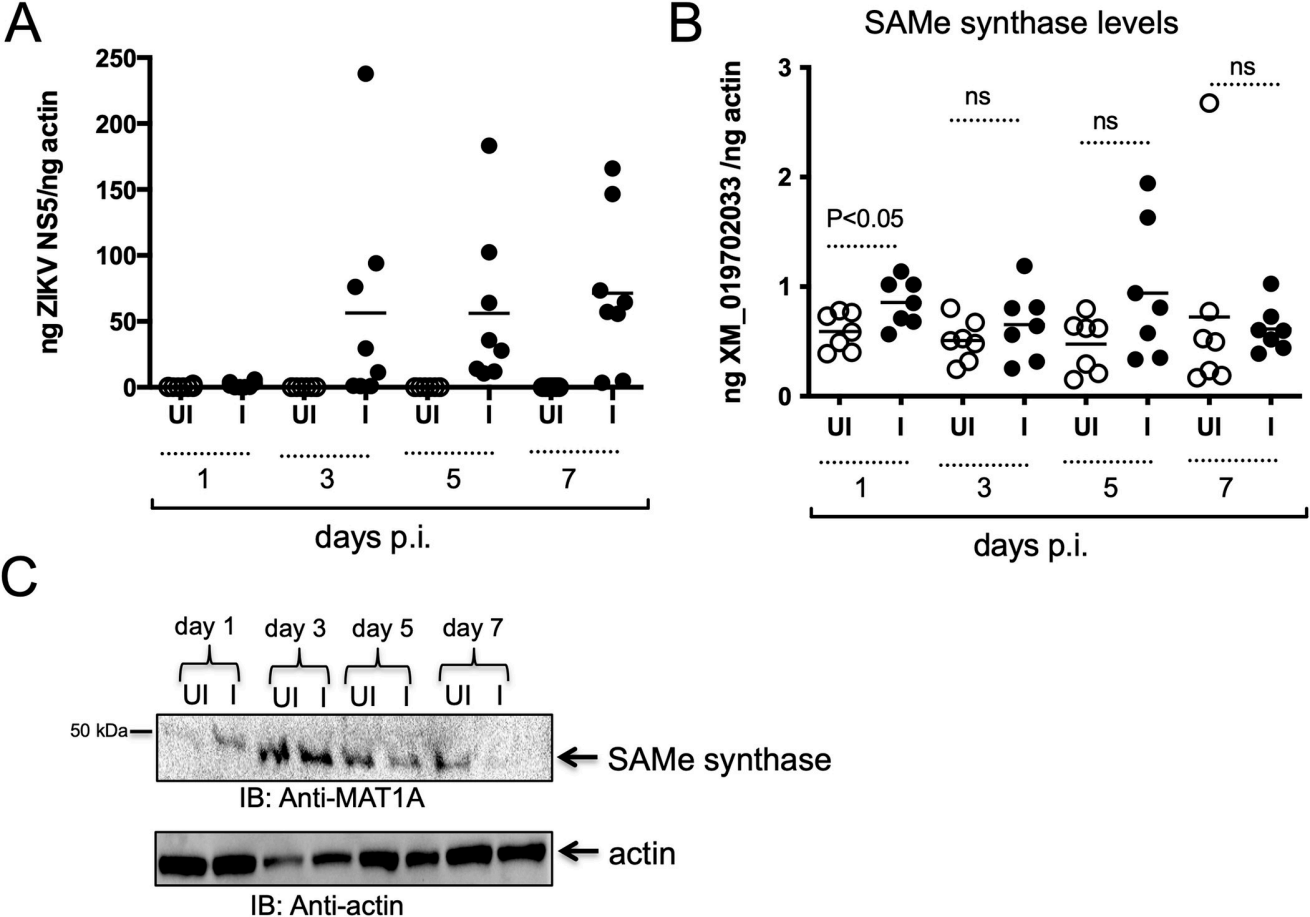
ZIKV upregulates SAMe synthase in mosquito cells. RT-qPCR analysis showing levels of ZIKV (A) and SAMe synthase (GenBank acc. no. XM_019702033) transcripts (B) in C6/36 cells at indicated time points (days 1, 3, 5, 7 p.i.). The levels of ZIKV NS5 and SAMe synthase transcripts were normalized to mosquito actin levels. In both A and B each circle indicates data from one independent culture well. Open circles indicate data from uninfected cells and closed circles indicate data from ZIKV-infected cells. Horizontal lines indicate mean of the values. P value from non-paired Student’s t-test is shown. ns indicates not significant. C) Immunoblotting analysis showing levels of SAMe synthase (alias name for MAT1A) at days 1, 3, 5, 7 p.i. Arrow indicates the SAMe synthase protein band. Actin levels serve as loading control. Full blot images are shown in S1 Fig. The mass of protein marker is indicated in kDa. In all panels UI indicates uninfected cells and I indicate ZIKV-infected cells.

We first assessed whether ZIKV (PRVABC59, the virulent Puerto Rico strain) infects and replicate in the *A*. *albopictus* C6/36 cell line. Reverse transcription quantitative PCR (RT-qPCR) analysis revealed that ZIKV readily infects C6/36 cells at multiplicity of infection (MOI) 1 with increased viral loads at days 3, 5, 7 post infection (p.i.) in comparison to day 1 p.i. (Fig 1A). *Aedes albopictus* genome encodes SAMe synthase-like gene (GenBank acc. no. XM_019702033). RT-qPCR analysis revealed that *A*. *albopictus* SAMe synthase transcripts were significantly (P<0.05) upregulated at earlier time point (day 1 p.i.) upon ZIKV infection in comparison to the uninfected controls (Fig 1B). However, no significant differences in SAMe synthase transcripts were evident between ZIKV-infected versus uninfected C6/36 cells at later time points (days 3, 5 and 7 p.i). of infection. No significant differences in the actin transcripts were noted between uninfected and ZIKV-infected C6/36 cells at tested time points (S1A Fig). Immunoblotting analysis with an anti-SAMe synthase antibody detected a band at size (~50 kDa) that was increased at day 1 p.i. in ZIKV-infected cells in comparison to the uninfected control (Figs 1C and S1B). Immunoblotting analysis of SAMe synthase levels at day 3 p.i. revealed no differences between ZIKV-infected cells in comparison to uninfected controls (Figs 1C and S1B). However, lower levels of SAMe synthase levels were noted at days 5 and 7 p.i. in ZIKV-infected cells in comparison to the uninfected controls (Figs 1C and S1B). Total actin levels served as loading controls (Figs 1C and S1C). Taken together, these results reveal that ZIKV modulates expression of SAMe synthase in mosquito cells.

### ZIKV infection increases SAMe levels in mosquito cells

**Fig 2:**
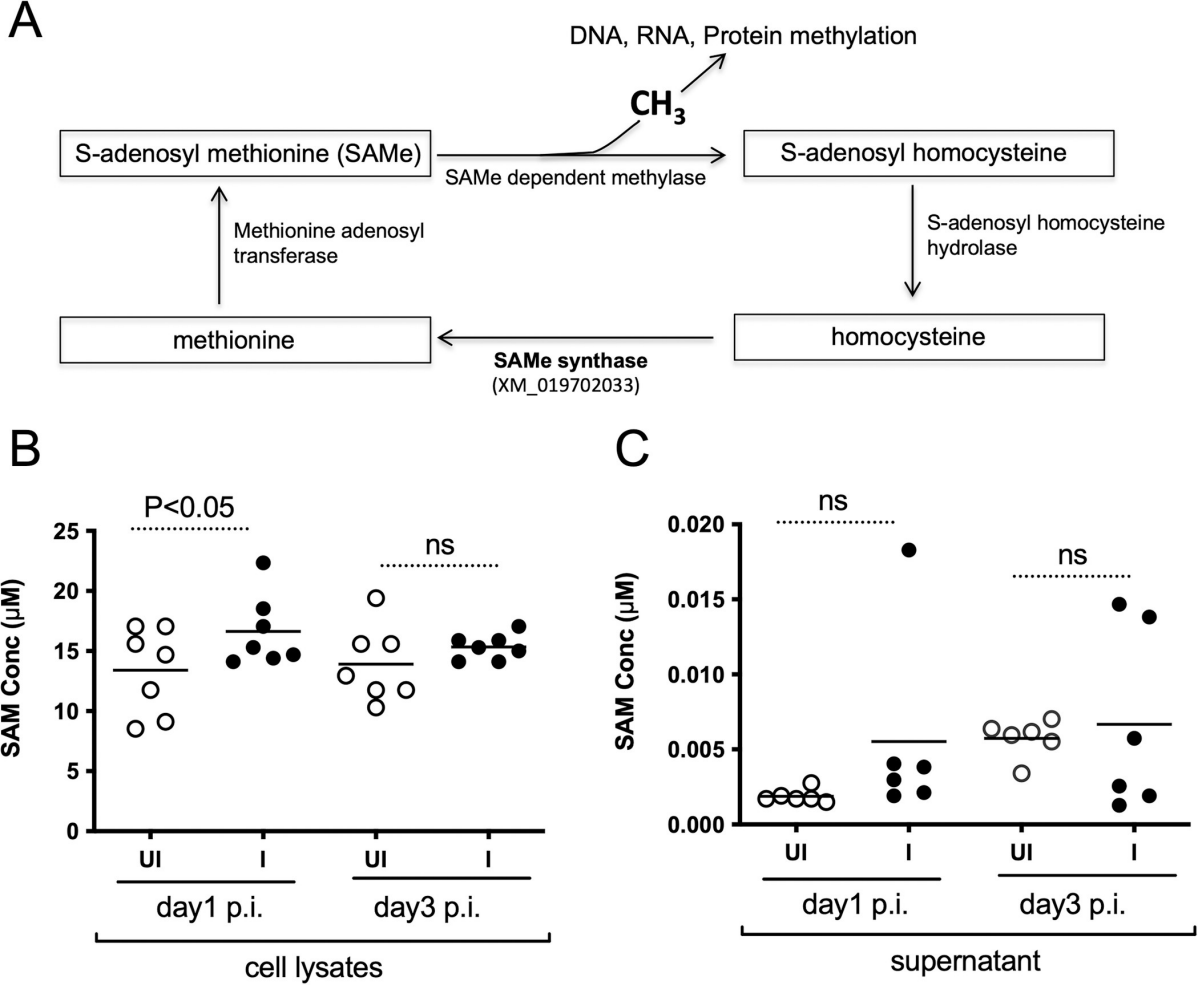
ZIKV induces SAMe levels in mosquito cells. A) Schematic representation of SAMe pathway. SAMe synthase participates in the synthesis of methionine from homocysteine. Methionine is then converted to SAMe by methionine adenosyl transferase. SAMe provides methyl group for methylation of nucleic acids and proteins and results in the formation of S-adenosyl homocysteine (SAH). SAH hydrolase converts SAH to homocysteine. Measurement of SAMe concentrations in uninfected (UI) and ZIKV-infected (I) cell lysates (B) and cell culture supernatants (C) is shown. Each circle represents SAMe levels from one independent assay well. Open circles indicate data from samples generated from uninfected cells and closed circle represents data from samples generated from ZIKV-infected cells. Horizontal lines indicate mean of the values. P value from non-paired Student’s t-test is shown. ns indicates not significant.

SAMe synthase is an important enzyme that participates in SAMe cycle [41] for the generation of methionine from homocysteine (Fig 2A). Methionine is further converted to SAMe by methionine adenosyltransferase (Fig 2A). SAMe acts as a main methyl donor for DNA, RNA and protein methylation (Fig 2A). The significant upregulation of SAMe synthase transcripts (Fig 1B) and increased protein at day 1 p.i. (Fig 1C) levels suggest that ZIKV could increase SAMe levels in mosquito cells at early time points of infection. We analyzed levels of SAMe in both uninfected and ZIKV-infected C6/36 cell lysates and cell culture supernatants collected at days 1 and 3 p.i.. A significant (P<0.05) increase in SAMe levels was noted in ZIKV-infected C6/36 cell lysates in comparison to uninfected controls at day 1 p.i. but not at day 3 p.i. (Fig 2B). However, no significant (P>0.05) difference was evident in cell culture supernatants (Fig 2C) between the two groups (uninfected or ZIKV-infected) at both tested time points (days 1 and 3 p.i). These results indicate a correlation between increased SAMe levels (Fig 2B) with increased SAMe-synthase RNA and protein levels (Fig 1B and 1C) at day 1 p.i. in mosquito cells upon ZIKV infection.

### Exogenous addition of SAMe increases ZIKV loads and histone methyl transferase transcript levels in mosquito cells

**Fig 3:**
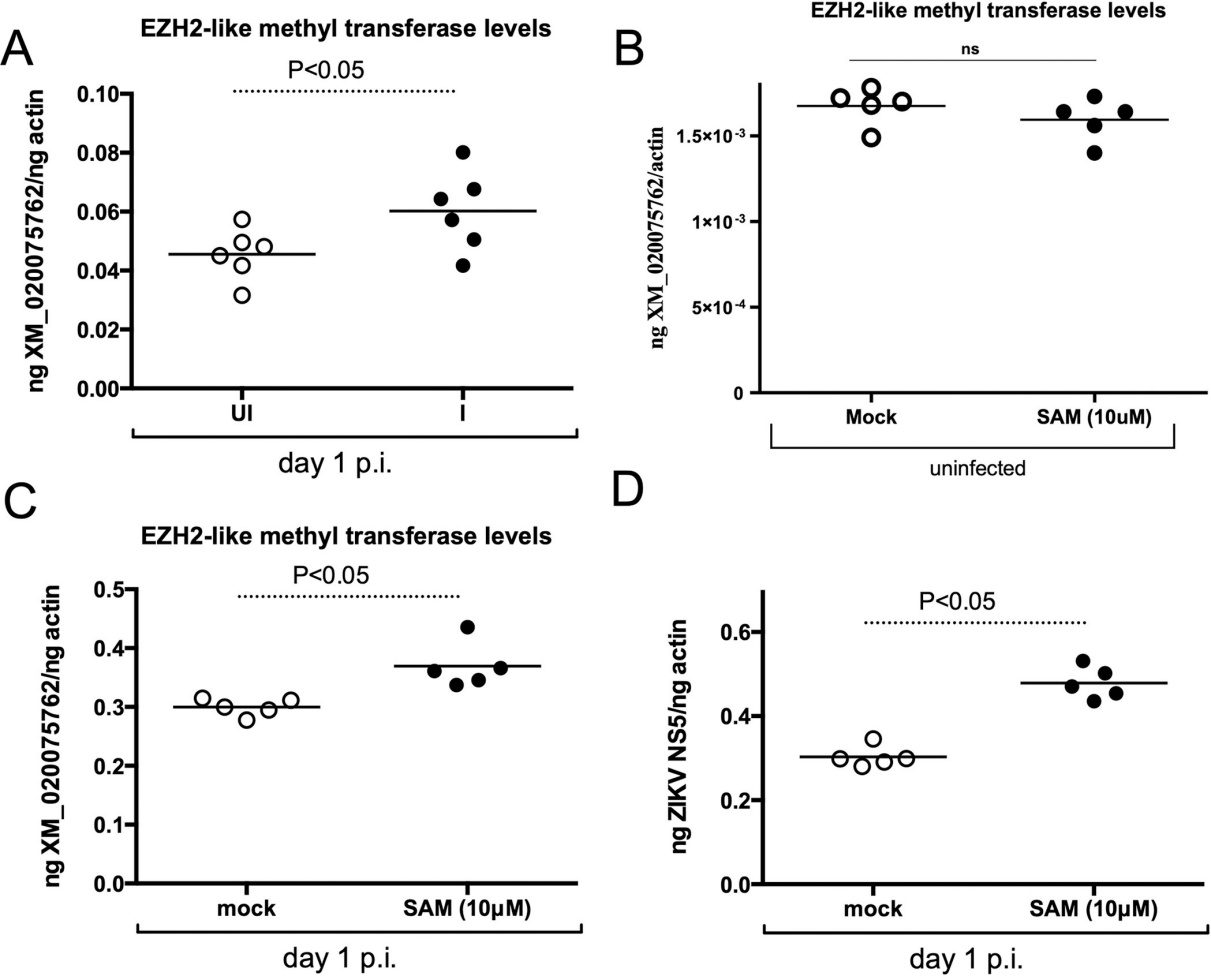
ZIKV and SAMe induces EZH2-like methyl transferase gene expression in mosquito cells. A) RT-qPCR analysis showing transcript levels of EZH2-like methyl transferase (GenBank acc. no. XM_020075762) in C6/36 cells at day 1 p.i. with ZIKV. Open circles indicate samples generated from uninfected cells and closed circle indicates samples generated from ZIKV-infected cells. RT-qPCR analysis showing levels of EZH2-like methyl transferase transcripts in uninfected (B), in ZIKV-infected (C) and viral loads (D) in C6/36 cells at day 1 p.i. treated with either mock (open circles) or SAMe (10 μM, closed circles). In all panels the levels of EZH2-like methyl transferase transcripts or ZIKV loads were normalized to mosquito actin levels. In all panels horizontal lines indicate mean of the values. P value from non-paired Student’s t-test is shown. NS indicates not significant.

The increased SAMe levels upon ZIKV infection of mosquito cells at day 1 p.i. (Fig 2B) suggests increased methylation of host or viral nucleic acids and/or proteins. SAMe is an important methyl donor for histone methylation [42]. Various methyl transferases methylate histone on several residues [42]. One among them is enhancer of Zeste homolog 2 (EZH2) methyltransferase that uses the methyl group from SAMe to trimethylate histone 3 lysine 27 (H3K27me3) [42,43]. *Aedes albopictus* encodes EZH2-like gene in its genome (GenBank acc. no. XM_020075762). RT-qPCR analysis revealed a significant (P<0.05) increase in mosquito EZH2-like gene transcripts in ZIKV-infected C6/36 cells in comparison to uninfected controls at day 1 p.i. (Fig 3A). We then analyzed whether treatment with exogenous SAMe (10 μM) has any effect on the EZH2-like gene transcripts in uninfected C6/36 cells. RT-qPCR analysis revealed no significant differences in the levels of EZH2-like gene transcripts between mock-treated and SAMe treated uninfected C6/36 cells (Fig 3B). Furthermore, treatment of ZIKV-infected mosquito cells with exogenous SAMe (10 μM) revealed no morphological changes in C6/36 cells in comparison to mock-treated control cells at day 1 p.i. (S2A Fig). However, a significant (P<0.05) increase in mosquito EZH2-like gene transcripts was noted in ZIKV-infected cells upon treatment with exogenous SAMe (10 μM) in comparison to mock-treated controls at day 1 p.i. (Fig 3C). Total actin transcripts levels were noted to be unaltered upon treatment with SAMe (S2B Fig). A significant (P<0.05) increase in ZIKV loads was noted in SAMe-treated C6/36 cells in comparison to mock-treated cells at day 1 p.i. (Fig 3D). Collectively, these results suggest that EZH2-like molecule and SAMe may have an important role in the ZIKV replication in mosquito cells.

### Comparison of *A*. *albopictus* histone H3 with other mosquitoes H3 orthologs

**Fig 4:**
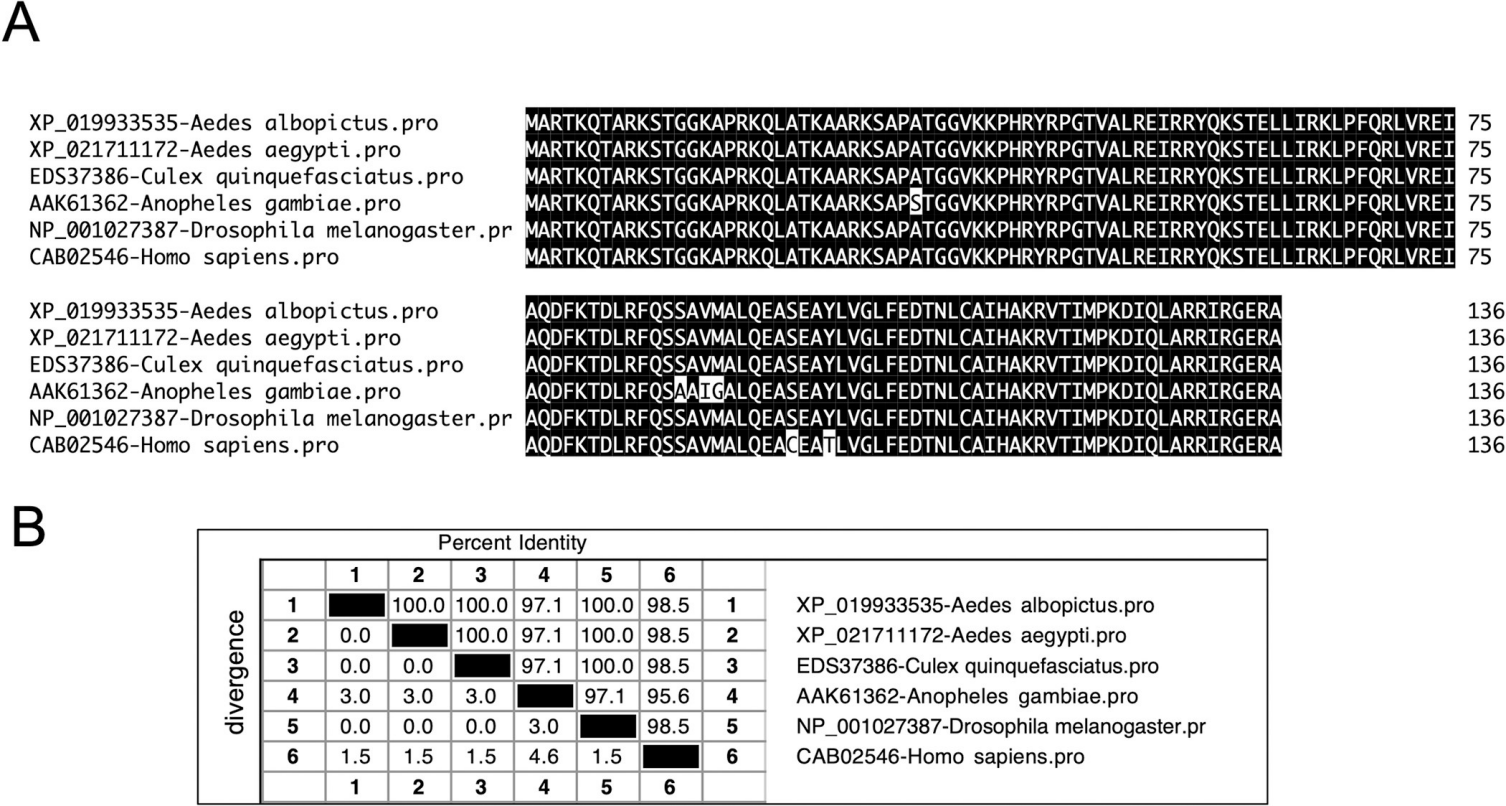
Alignment and phylogenetic analysis of *A*. *albopictus* histone H3. A) The *A*. *albopictus* H3 amino acid sequence alignment (with other mosquito, fly and human orthologs) using ClustalW program in DNASTAR (Lasergene Genomics Suite) is shown. Residues that match are shaded in black color. GenBank accession numbers for *A*. *albopictus*, *A*. *aegypti*, *C*. *quinquefasciatus*, *A*. *gambiae*, *D*. *melanogaster* and *H*. *sapiens* sequences are shown. Total length and percent identities of the amino acid sequences are provided at one end of each sequence. B) The percent identity (horizontally above black-boxed diagonal) and divergence (vertically below black-boxed diagonal) for *A*. *albopictus* H3 amino acid sequence in comparison to other H3 sequences is shown. Sequence distances data was generated based on the ClustalW alignment of the sequences.

We first performed bioinformatics analysis to determine *A*. *albopictus* histone H3 protein relatedness with other H3 proteins. GenBank annotated primary amino acid sequences of *A*. *albopictus* histone H3 (GenBank acc. no. XP-019933535), *A*. *aegypti* histone H3 (GenBank acc. no. XP_021711172), *Culex quinquefasciatus* histone H3 type 2 (GenBank acc. no. EDS37386), *Anopheles gambiae* histone 3A (GenBank acc. no. AAK61362), *Drosophila melanogaster* histone H3 (GenBank acc. no. NP_001027387) and *Homo sapiens* histone H3 (GenBank acc. no. CAB02546) were downloaded and analyzed in DNASTAR. ClustalW alignment of all analyzed sequences revealed a high degree of conservation across the entire amino acid sequence (Fig 4A). *Aedes albopictus* H3 shared 100% identity with H3 from *A*. *aegypti*, *C*. *quinquefasciatus* and *D*. *melanogaster*, 97.1% identity with *A*. *gambiae* and 98.5% identity with H3 from *H*. *sapiens* (Fig 4B). The lysine residue involved in H3K27me3 was present in all the H3 sequences including *A*. *albopictus* (Fig 4A).

### ZIKV induces H3K27me3 methylation in mosquito cells

**Fig 5:**
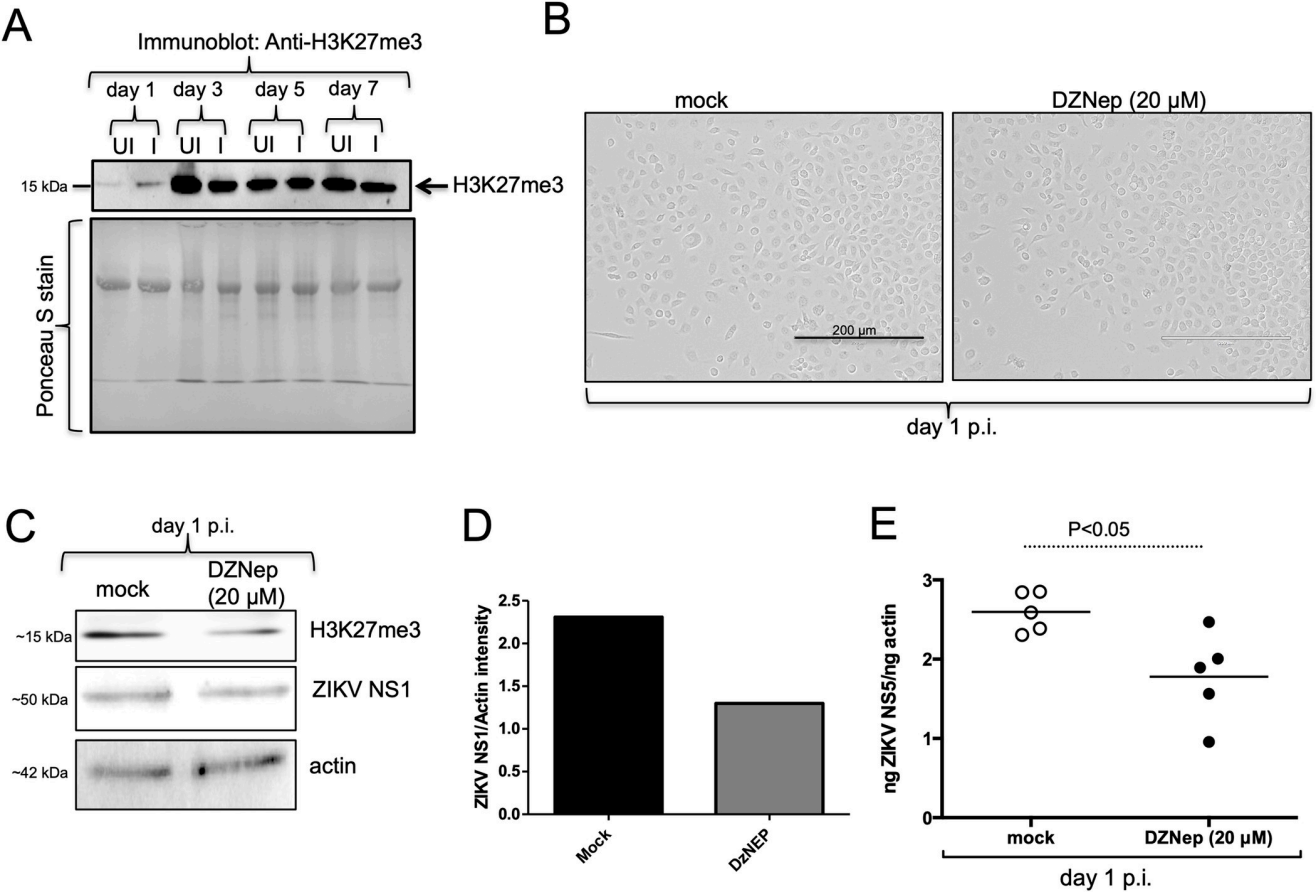
Treatment with DZNep (EZH2-methyl transferase inhibitor) affects H3K27me3 methylation and ZIKV burden in mosquito cells. A) Immunoblotting analysis showing levels of H3K27me3 in uninfected or ZIKV-infected cells at days 1,3,5,7 p.i. Arrow indicates the H3K27me3 protein band at ~15 kDa. Ponceau S-stained membrane image serve as loading control. The mass of protein marker is indicated in kDa. In both panels UI indicates uninfected cells and I indicate ZIKV-infected cells. B) Phase contrast microscopic images of ZIKV-infected C6/36 cells treated with either mock or DZNep (20 μM) is shown. Scale bar indicates 200 μm. C) Immunoblotting analysis showing levels of H3K27me3, ZIKV NS1 and actin in mock or DZNep-treated ZIKV-infected cells at day 1 p.i. Actin levels serve as loading control. Full blot images are shown in S3 and S5 Figs. The mass of protein marker is indicated in kDa. D) Densitometry analysis of ZIKV NS1 levels noted in the immunoblot shown in panel C. E) RT-qPCR analysis showing viral loads in mock- (open circles) or DZNep (20 μM, closed circles)-treated ZIKV-infected C6/36 cells at day 1 p.i. The ZIKV loads were normalized to mosquito actin levels. Horizontal line indicates mean of the values. P value from non-paired Student’s t-test is shown.

H3K27me3 is often associated with down regulation of gene expression by creating heterochromatin and restricting access of transcription factors to target genes [42–44]. The increased mosquito EZH2-like gene transcripts upon ZIKV infection at day 1 p.i. suggests increase in H3K27me3 levels. Total protein extracts from ZIKV-infected or uninfected C6/36 cells generated from days 1, 3, 5 and 7 p.i. were processed for immunoblotting analysis with H3K27me3 specific antibody. An increase in the H3K27me3 levels was noted in ZIKV-infected C6/36 cells in comparison to uninfected controls at day 1 p.i. (Figs 5A and S3). However, no differences in H3K27me3 levels were noted between ZIKV-infected cells and uninfected controls (Figs 5A and S3) at later time points (days 3, 5, 7 p.i.).

### Treatment with 3-Deazaneplanocin A hydrochloride (DZNep), an inhibitor of EZH2 inhibits H3K27me3 and ZIKV replication in mosquito cells

A study has reported that treatment of mammalian cells with DZNep, a carbocyclic analog of adenosine, depletes cellular levels of EZH2 and inhibits H3K27me3 [45]. We first treated C6/36 cells with DZNep at 5 μM concentration followed by ZIKV infection for 24 h. Microscopic analysis revealed no cytotoxic effects of DZNep on C6/36 cells at 5 μM concentration (S4A Fig). In addition, RT-qPCR analysis revealed no significant (P>0.05) difference in ZIKV loads in C6/36 cells upon treatment with 5 μM DZNep in comparison to the mock control (S4B Fig). We then treated C6/36 cells with DZNep at 20 μM concentration followed by ZIKV infection for 24 h. Microscopic analysis revealed no cytotoxic effects of DZNep on C6/36 cells at 20 μM concentration (Fig 5B). Immunoblotting analysis revealed reduced H3K27me3 levels in ZIKV-infected C6/36 cells treated with 20 μM DZNep in comparison to mock-treated controls (Figs 5C and S5A). In addition, we noted approximately two-fold reduction in the viral loads upon treatment to ZIKV-infected C6/36 cells treated with 20 μM DZNep (Figs 5C, 5D and S5B). Levels of actin served as loading control in the immunoblotting analysis (Figs 5C and S5C). Furthermore, RT-qPCR revealed a significant (P<0.05) reduction in ZIKV loads in C6/36 cells upon treatment with 20 μM DZNep in comparison to the mock control (Fig 5E). Collectively, these results reveal that histone methylation mediated by EZH2 methyl transferase and SAMe is important for ZIKV replication in mosquito cells.

## Discussion

**Fig 6:**
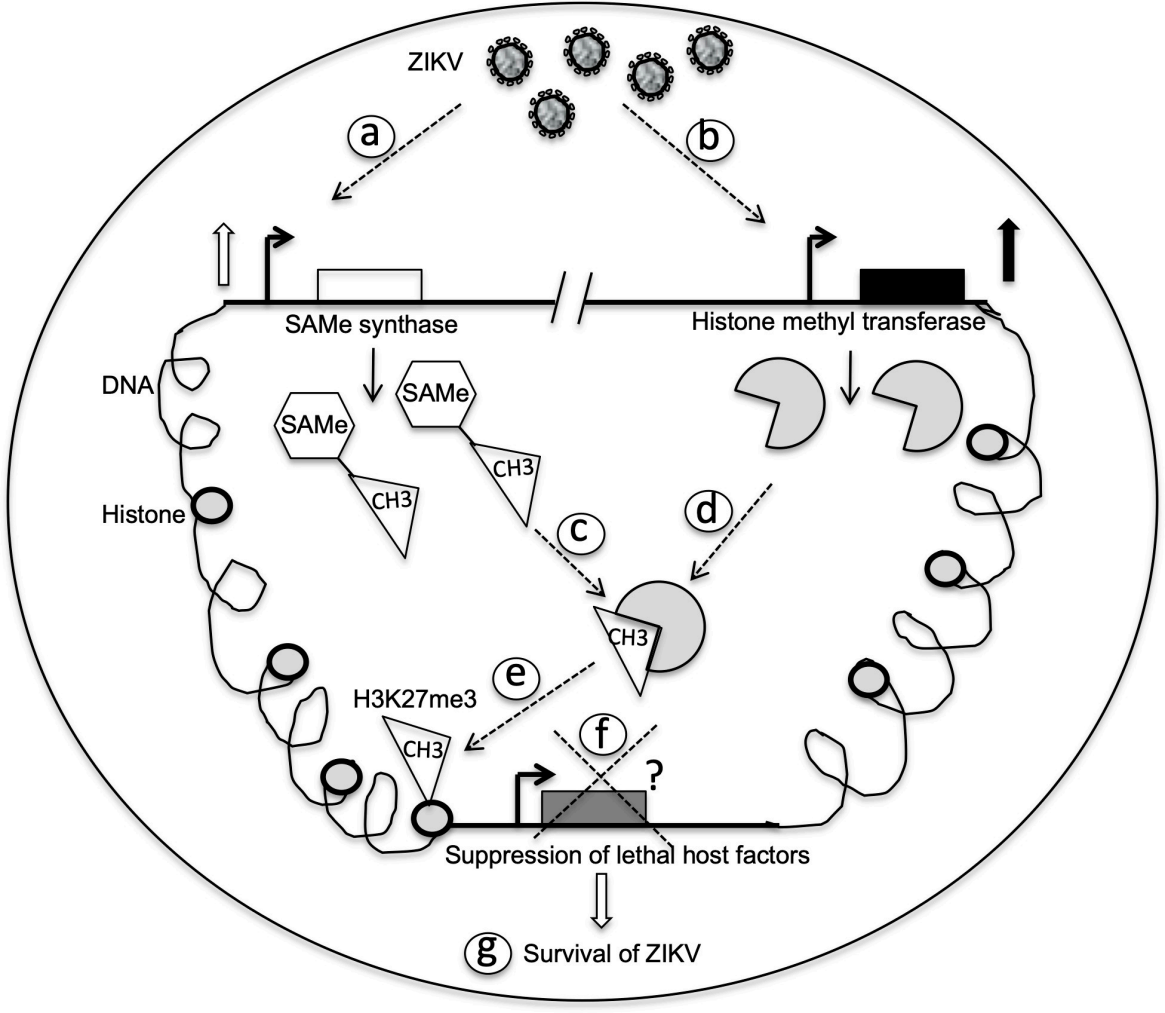
Schematic model showing roles of SAMe and H3K27me3 signaling in ZIKV survival in mosquito cells. ZIKV up-regulates SAMe-synthase (a) and EZH2-methyl transferase (b) gene expression upon entry into mosquito cells (a). The increased levels of SAMe (c) and EZH2-methyl transferases (d) lead the later enzyme to acquire methyl group for transfer to histone 3 (H3). e) EZH2-methyl transferase methylate H3 that may subsequently lead to transcriptional silencing (f) of lethal host factors that inhibit ZIKV replication in mosquito cells. g) The absence of lethal host factors for viral replication leads ZIKV successfully to replicate and establish persistent infection in mosquito cells.

The emergence of ZIKV infections through infected mosquito bites suggests immediate need for many studies that delineate strategies to target ZIKV-mosquito interactions. Even though *A*. *albopictus* is not considered as the primary vector for ZIKV, any studies related to this species could be directly translated in understanding interactions of ZIKV with other *Aedes* species. In this study, we provide evidence that ZIKV modulates arthropod SAMe cycle and histone methylation for its survival in *A*. *albopictus* cells. Our study provides a model (Fig 6) to understand modulation of H3K27me3 methylation in ZIKV survival in mosquito cells. Upon entry into mosquito cells and at the initial phase of infection, ZIKV upregulates SAMe synthase to produce more of SAMe (Fig 6). Availability of increased SAMe may facilitate methylation of ZIKV RNA as well as mosquito histone (Fig 6). In addition, up-regulation of EZH2 transcripts at the initial phase of ZIKV infection (Fig 6) could facilitate increased transfer of methyl groups from SAMe to enhance H3K27me3. The increased H3K27me3 levels may aid in the repression of host factors that inhibit ZIKV replication in mosquitoes. These factors could include molecules in the mosquito RNAi, JAK/STAT, Toll, IMD, and MAPK pathways [46].

Methylation is an important post-translational modification on nucleic acids and proteins that is critical for various cellular functions [47]. Flavivirus positive strand RNA genome contains a 5’ terminal cap 1 structure (m^7^GpppAmG). The viruses belonging to flavivirus group encode their own methyl transferase, located at the N-terminus region of NS5 protein that catalyzes methylation during capping of viral RNA genome [39]. A study reported that an analog for SAMe intrudes the RNA-cap binding site of ZIKV methyl transferase [48]. Our observation of increased ZIKV loads upon treatment with exogenously added SAMe clearly suggests importance of this molecule as a methyl donor during viral replication. A study has suggested that SAMe could be transported via the NA^+^-independent nucleoside carrier systems [49]. In our tested conditions, we believe that exogenous SAMe was transported in a similar way into the mosquito cells that facilitates ZIKV replication. The upregulation of SAMe synthase transcripts at initial phase (day 1 p.i.) of infection (Fig 1B) clearly provides further evidence on the role of SAMe in viral replication in mosquito cells. At the initial phase of ZIKV infection, the increased levels of SAMe synthase could efficiently convert homocysteine to methionine. The increased levels of methionine could then lead to the synthesis of high levels of SAMe. SAMe could serve as a methyl donor for both RNA and proteins [50]. Therefore, it is not surprising to hypothesize that ZIKV could increase SAMe levels to methylate its own RNA and lead to mosquito histone 3 methylation. SAMe is converted to S-adenosyl homocysteine (SAH) by SAMe-dependent methylase. SAH is an inhibitor of reactions mediated by SAMe [51]. In addition, SAH is reported to exhibit cytotoxic effects in human cells [52]. The observation of lower levels of SAMe synthase (MAT1A) protein in ZIKV-infected mosquito cells at later time point (eg. day 7 p.i.) suggests that ZIKV may lower SAMe levels to affect SAH synthesis. We believe that lower SAH levels could facilitate better cell survival and facilitate ZIKV infection for extended time in the vector host cells.

SAMe-dependent methyl transferases are classified into three classes based on their structures: PMTs (protein methyl transferases) that contain seven-strand twisted beta-sheet structure, SET (SuVar3-9, enhancer of Zeste, Trithorax) domain containing lysine methyl transferases and membrane-associated methyl transferases [53]. EZH2 belongs to SET 1 family of methyl transferases that tri-methylate histone H3 (H3K27me3) to cause transcriptional silencing [54]. EZH2 localizes with the promoter region of HIV-1 proviruses in latently infected Jurkat T cell that contained H3K27me3 [55]. The knockdown of EZH2 or treatment of these cells with DZNep reactivates proviruses [55]. EZH2 has also been implicated in the regulation of the lytic-latency cycles of HCV [55]. In addition to these studies, a report has shown that treatment of mammalian cells with EZH2/1 inhibitor induces antiviral state and suppress infection by diverse viral pathogens including ZIKV [56]. The observation of decreased mosquito H3K27me3 levels and viral loads (Fig 5) upon treatment with DZNep clearly suggests that ZIKV modulates EZH2-mediated H3K27me3 levels for silencing host factors that potentially restrict viral replication in arthropod cells. The increased expression of EZH2 transcripts and H3K27me3 methylation levels at the initial phase of ZIKV infection of mosquito cells further supports the observation noted with DZNep treatment. The observation of no differences in the H3K27me3 levels at later stage of infection suggests that EZH2-mediated H3K27me3 methylation is important for early phase of ZIKV infection. Our study in conjunction with the findings from Arbuckle et al., [56] suggests that ZIKV may use EZH2-mediated H3K27me3 to suppress antiviral genes in arthropod and mammalian cells. A high degree of conservation between mosquito and human EZH2 amino acid sequence (S6 Fig) further supports this view.

In summary, our study reports that ZIKV induces expression of SAMe synthase and EZH2 histone methyl transferase that led to increased H3K27me3 methylation at the initial phase of infection possibly to repress the host factors that inhibit virus replication in mosquito cells. The anti-viral responses in the host cells could be observed for prolonged periods of time. Therefore, future studies could explore whether SAMe synthase-EZH2-mediated H3K27me3 levels are critical for suppressing these responses at different stages of infection. The findings from our study on the inhibition of virus replication upon treatment with EZH2 inhibitor suggests therapeutic potential for this inhibitor to block ZIKV replication in arthropod cells. Characterization of vector molecules such as these that participate in SAMe cycle or histone methylation could lead for the development of anti-vector vaccines [57] to block ZIKV transmission from mosquitoes to humans.

## Methods

### Cell culture, reagents and infection

Mosquito cells were cultured as described previously [58,59]. Briefly, mosquito (C6/36) cell line was purchased from American Type Culture Collection (ATCC). C6/36 cells were plated in a 12 well plate at a density of 1 x 10^5^ cells per well in complete MEM medium containing 10% FBS, L-glutamine and Pen/Strep and kept at 30° C with 5% CO2. All infections were performed with ZIKV strain PRVABC59 (MOI = 1). For ZIKV infection kinetics experiment, 1 x 10^5^ C6/36 cells were plated and infected with ZIKV at 24 h post plating followed by collection of cells at indicated time points (days 1, 3, 5, 7 p.i.) and processed for RNA or protein extraction. ZIKV, PRVABC59 strain (Catalog number NR-50240) was obtained from BEI resources and propagated in Vero (African Green Monkey kidney) cell line as per the instructions from the distributor and viral titers were determined based on the plaque assays performed on Vero cells.

### RNA extraction, cDNA synthesis and RT-qPCR analysis

Total RNA from mosquito cells was generated using the Aurum Total RNA mini kit (Bio-Rad, USA) following the manufacturer’s instructions. The isolated RNA was converted to cDNA using iScript cDNA synthesis kit (BioRAD, USA) and processed for RT-qPCR as described [59–63]. S1 Table contains sequences for the oligonucleotides used in this study. We used 10 pmol of each primer for each RT-qPCR reaction. In RT-qPCR analysis, mosquito *beta-actin* was used as an internal control to normalize the amount of template in each reaction. RT-qPCR was performed using CFX96 machine (BioRad, USA) and iQ-SYBR Green Supermix (BioRad, USA). ZIKV burden in mosquito cells was quantified from total RNA extracts isolated from infected or uninfected cells. The RT-qPCR reactions were performed for each gene with its own standards (1ng/ul to 0.000001ng/ul). The average value of each well (in ng) was normalized to actin levels measured in similar way with its own standard (1ng/ul to 0.000001ng/ul). The ratio of gene/actin value or NS5/actin values were considered to plot the scatter plot.

### Immunoblotting

Immunoblotting was performed as described [58,59,64]. Briefly, 1 x 10^5^ mosquito cells were seeded in 12 well-plates for overnight incubation and infected with 1 MOI ZIKV the next day. Cells were collected at different time points (days 1, 3, 5, 7 p.i.) and processed for total protein extraction. Cell pellet was washed twice with 1 X PBS and re-suspended in RIPA lysis buffer. Cells were homogenized and total protein amounts in each sample were measured using BCA kit (Pierce/ThermoScientific). Total protein lysates (25–35 μg) were separated on 12% SDS-PAGE gels by gel electrophoresis and transferred to nitrocellulose membrane. The blots were then blocked with 5% milk buffer and treated with primary antibodies, rabbit monoclonal anti-MAT1A (Abcam, USA) or mouse monoclonal anti-H3K27me3 (Abcam, USA) followed by secondary anti-rabbit HRP conjugated (SantaCruz Inc., USA) or anti-mouse HRP conjugated (SantaCruz Inc., USA) antibodies, respectively. Ponceau S stained images showing total protein profiles were used as loading controls. Antibody binding was detected with WesternBright ECL kit (Advansta, BioExpress). Blots were imaged using Chemidoc MP imaging system and processed using Image Lab software obtained from the manufacturer (BioRad).

### Measurement of SAMe in mosquito cells

SAMe levels from uninfected or ZIKV-infected cells were measured by using Bridge-It-S-adenosyl Methionine fluorescence assay kit (Mediomics LLC, USA) and following manufacturers’ protocol. Briefly, 5 x 10^4^ mosquito cells were seeded in 12 well-plates for overnight incubation and infected with 1 MOI ZIKV the next day. Cells and culture supernatants were collected at two time points (day 1 and 3 p.i). Cell pellets were washed twice with 1 x PBS and treated with CM buffer (included in the kit) for cell lysis and release of SAMe. The collected supernatant was processed as per manufacturer instructions. The cell culture supernatants were diluted with Buffer S (included in the kit) and processed for SAMe determination. The fluorescence intensity for SAMe levels was measured using TECAN fluorescence microplate reader (TECAN, USA) with settings excitation at 485 nm and emission at 665 nm. A standard curve was generated using known amounts of SAMe (provided in the kit) and concentrations of SAMe from cells or supernatants was measured relative to the standard curve. Microscopy (phase contrast) analysis was performed using the EVOS Fluorescence System (Invitrogen/ThermoScientific) as described [65,66].

### SAMe or DZNep treatment studies

For exogenous SAMe or DZNep treatment experiments, 1 x 10^5^ mosquito cells were seeded in 12 well plates for overnight incubation and treated with 10 mM SAMe (Mediomics LLC, USA) to a 10 μM final concentration or with 1mM DZNep (Abcam, USA) to 5 μM or 20 μM final concentration the next day for four hours followed by ZIKV infection (MOI = 1). Equal volume of 10 mM 2-mercaptoethanol was used as mock solution for SAMe-treatment studies or equal volume of distilled water was used as mock for DZNep-treatment studies. Cells were collected at day 1 p.i. and processed for RNA extraction followed by RT-qPCR to determine EZH2 gene expression or ZIKV loads.

### GenBank accession numbers used in this study

Following are the GenBank accession numbers used in this study: *A*. *albopictus* S-adenosylmethionine (SAMe) synthase-like gene (GenBank acc. no. XM_019702033), *A*. *albopictus* encodes EZH2-like gene in its genome (GenBank acc. no. XM_020075762), *A*. *albopictus* histone H3 (GenBank acc. no. XP-019933535), *A*. *aegypti* histone H3 (GenBank acc. no. XP_021711172), *Culex quinquefasciatus* histone H3 type 2 (GenBank acc. no. EDS37386), *Anopheles gambiae* histone 3A (GenBank acc. no. AAK61362), *Drosophila melanogaster* histone H3 (GenBank acc. no. NP_001027387) and *Homo sapiens* histone H3 (GenBank acc. no. CAB02546).

### Statistics

GraphPad Prism6 software and Microsoft Excel 2016 were used to analyze statistical significance in the data sets. The non-paired Student t-test was performed to compare two means. The comparison was performed with two groups in all the data analysis. P values of <0.05 were considered significant in all analysis. Horizontal lines in graphs represent mean of the readings. P values are indicated at the relevant places in the figures. Non-significant differences are indicated with “ns”.

## Supporting information

S1 Fig SAMe synthase levels in mosquito cells at different days upon ZIKV infection. A) RT-qPCR analysis showing levels of actin normalized to total RNA in uninfected or ZIKV-infected C6/36 cells at days 1, 3, 5, 7 p.i. Full length immunoblot image showing levels of MAT1A (alias name for SAMe synthase) (B) and actin (C) at days 1, 3, 5, 7 p.i. Arrow indicates the SAMe synthase (B) or actin (C) protein bands. The mass of protein marker is indicated in kDa. The expected band size around 50 kDa is indicated with an arrow. The band around 37 kDa could be a cleaved form/spliced variant/partially translated product of SAMe synthase. In all panels UI indicates uninfected and I indicate ZIKV-infected cells.
(TIF)

S2 Fig Exogenous treatment with SAMe on C6/36 cells. A) Phase contrast microscopic images of ZIKV-infected C6/36 cells treated with either mock or SAMe (10 μM) is shown. Scale bar indicates 200 μm. B) RT-qPCR analysis showing levels of actin normalized to total RNA upon treatment of ZIKV-infected C6/36 cells with either mock or SAMe (10 μM). ns indicates not significant.
(TIF)

S3 Fig H3K27me3 levels in C6/36 cells at different days upon ZIKV infection. Full-length immunoblot image showing levels of H3K27me3 in uninfected or ZIKV-infected cells at days 1,3, 5, 7 p.i. Arrow indicates the H3K27me3 band at ~15 kDa. The mass of protein marker is indicated in kDa. In both panels UI indicates uninfected cells and I indicates ZIKV-infected cells.
(TIF)

S4 Fig Treatment with DZNep (EZH2-methyl transferase inhibitor) at 5 μM has no effect on ZIKV replication in mosquito cells. A) Phase contrast microscopic images of ZIKV-infected C6/36 cells treated with either mock or DZNep (5 μM) is shown. Scale bar indicates 200 μm. B) RT-qPCR analysis showing viral loads in mock- (open circles) or DZNep (5 μM, closed circles)-treated ZIKV-infected C6/36 cells at day 1 p.i.. The ZIKV loads were normalized to mosquito actin levels. Horizontal line indicates mean of the values and P value from non-paired Student’s t-test is shown.
(TIF)

S5 Fig H3K27me3 levels in C6/36 cells at different days upon ZIKV infection. Full-length immunoblot images showing levels of H3K27me3 (A), ZIKV NS1 (B) or actin (C) in mock or DZNep-treated ZIKV-infected cells at days 1 p.i. Arrow indicates the H3K27me3 band at ~15 kDa (A), ZIKV NS1 band around 50 kDa and actin band around 42 kDa (C). The mass of protein marker is indicated in kDa.
(TIF)

S6 Fig Amino acid sequence alignment of *A*. *albopictus* EZH2-methyl transferase with other orthologs. The *A*. *albopictus* EZH2 methyl transferase amino acid sequence alignment with *A*. *aegypti* and human orthologs using ClustalW program in DNASTAR (Lasergene Genomics Suite) is shown. Residues that match are shaded in black color. GenBank accession numbers for *A*. *albopictus*, *A*. *aegypti* and *H*. *sapiens* sequences are shown. Total length and percent identities of the amino acid sequences are provided at one end of each sequence.
(TIF)

S1 Table Oligonucleotides used in this study. Oligonucleotides used in this study are listed in this table.
(PDF)

S1 File (XLSX)
