# Supplementary figures and images for: Zika virus modulates arthropod histone methylation for its survival in mosquito cells
Source: PLoS One. 2025 Feb 13;20(2):e0319290. doi: 10.1371/journal.pone.0319290 (PMC11824992; doi:10.1371/journal.pone.0319290)

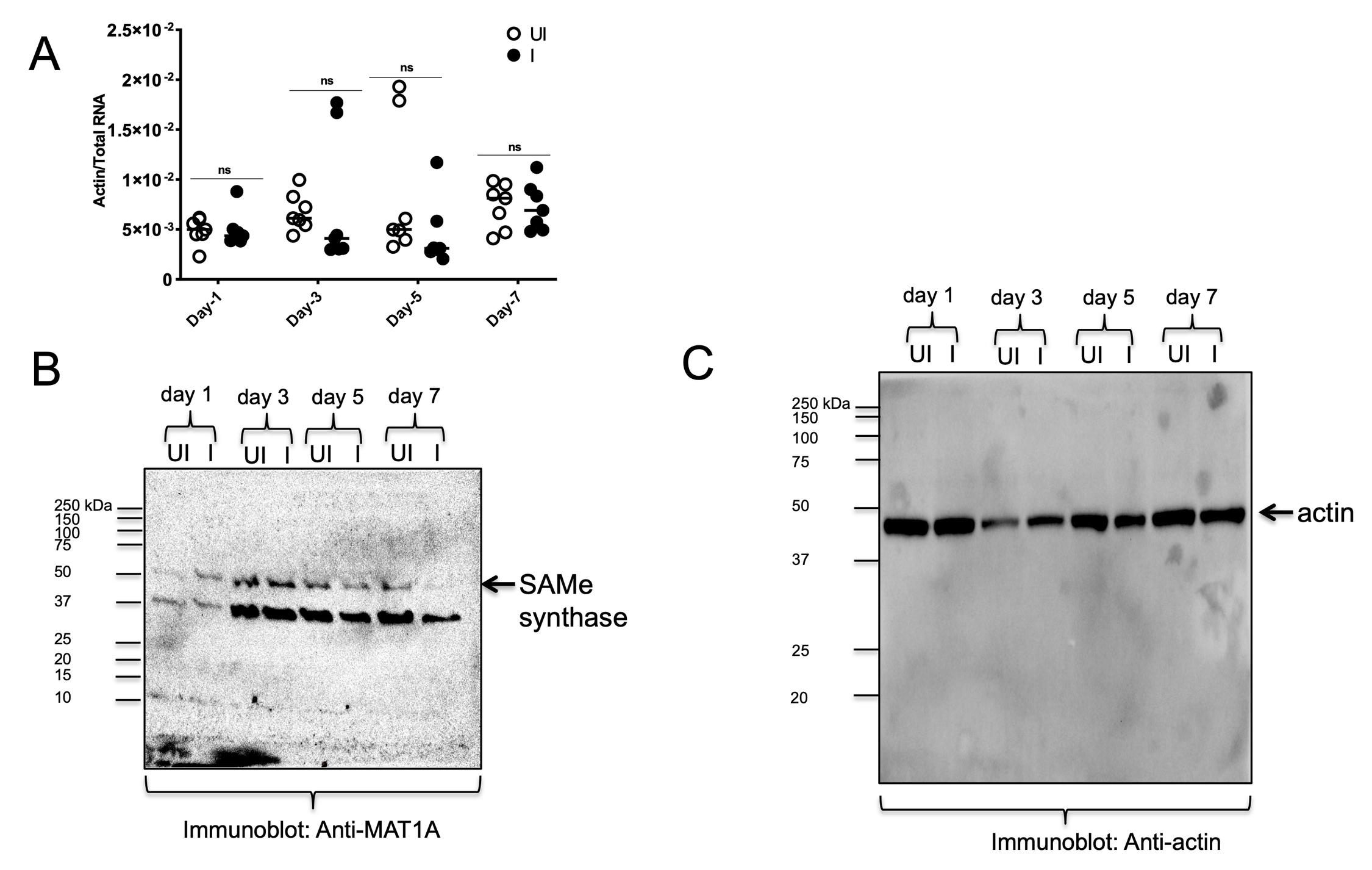

Supplement: S1 Fig — A) RT-qPCR analysis showing levels of actin normalized to total RNA in uninfected or ZIKV-infected C6/36 cells at days 1, 3, 5, 7 p.i. Full length immunoblot image showing levels of MAT1A (alias name for SAMe synthase) (B) and actin (C) at days 1, 3, 5, 7 p.i. Arrow indicates the SAMe synthase (B) or actin (C) protein bands. The mass of protein marker is indicated in kDa. The expected band size around 50 kDa is indicated with an arrow. The band around 37 kDa could be a cleaved form/spliced variant/partially translated product of SAMe synthase. In all panels UI indicates uninfected and I indicate ZIKV-infected cells. (TIF) [file pone.0319290.s001.tif]

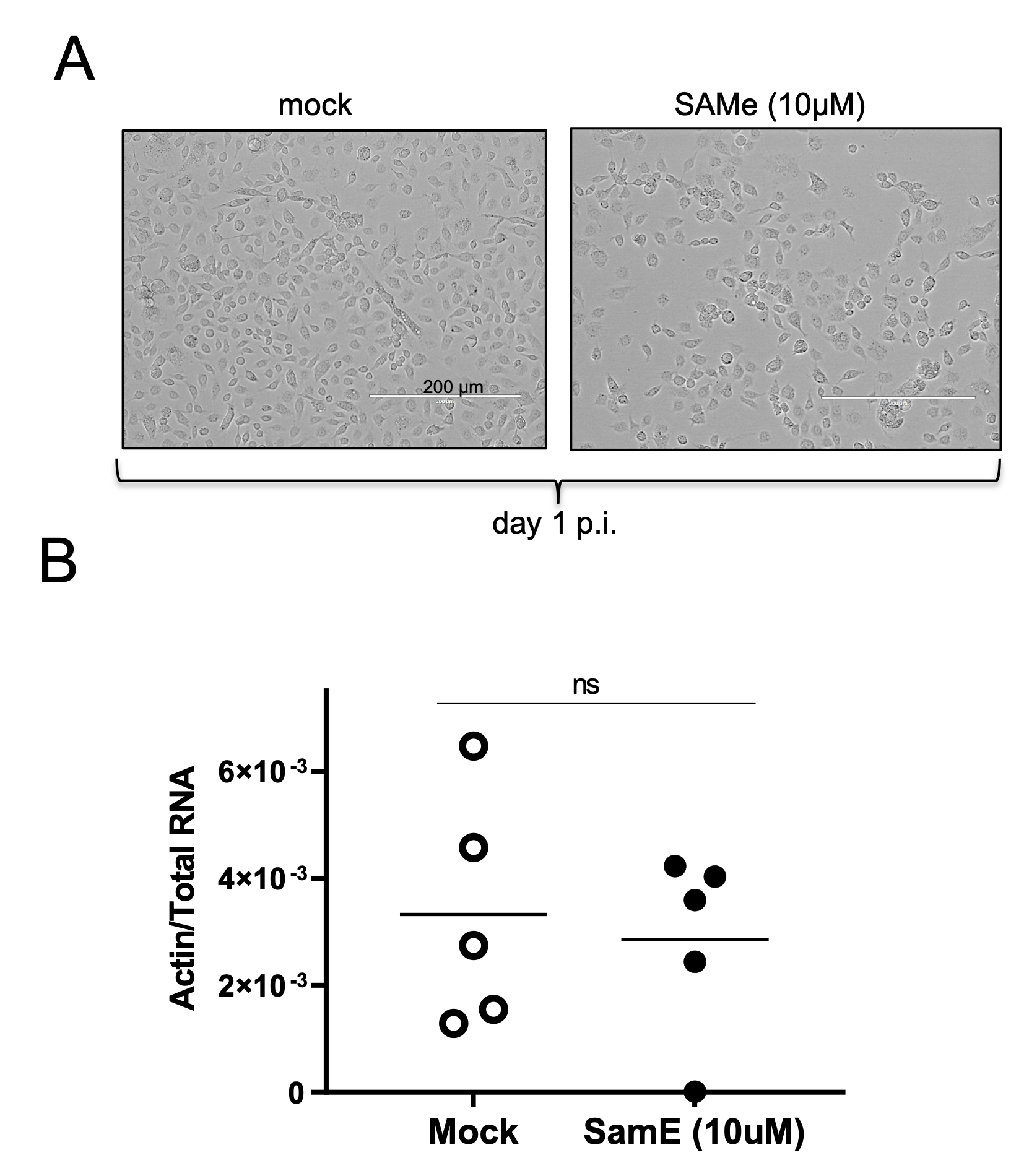

Supplement: S2 Fig — A) Phase contrast microscopic images of ZIKV-infected C6/36 cells treated with either mock or SAMe (10 μM) is shown. Scale bar indicates 200 μm. B) RT-qPCR analysis showing levels of actin normalized to total RNA upon treatment of ZIKV-infected C6/36 cells with either mock or SAMe (10 μM). ns indicates not significant. (TIF) [file pone.0319290.s002.tif]

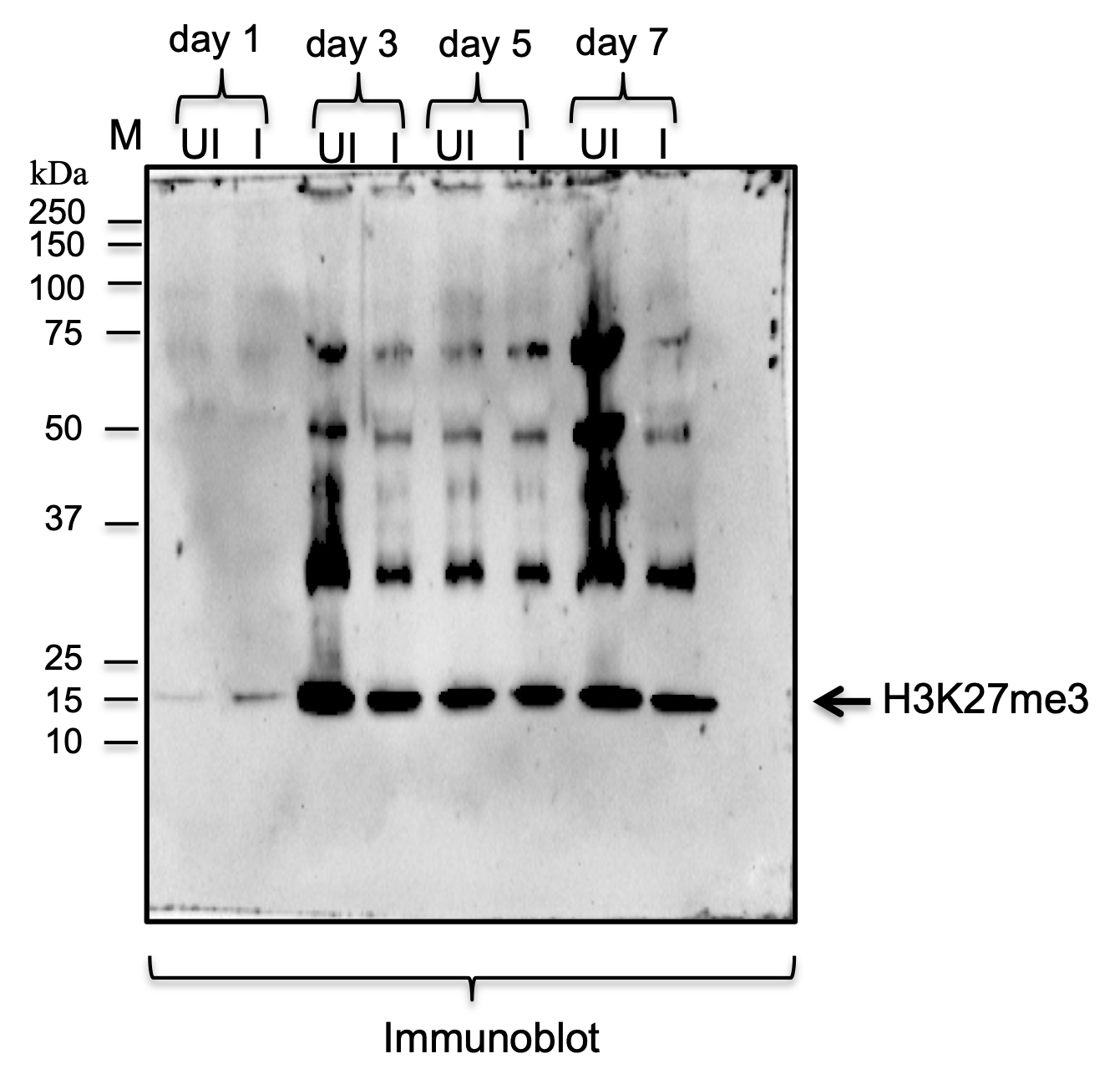

Supplement: S3 Fig — Full-length immunoblot image showing levels of H3K27me3 in uninfected or ZIKV-infected cells at days 1,3, 5, 7 p.i. Arrow indicates the H3K27me3 band at ~15 kDa. The mass of protein marker is indicated in kDa. In both panels UI indicates uninfected cells and I indicates ZIKV-infected cells. (TIF) [file pone.0319290.s003.tif]

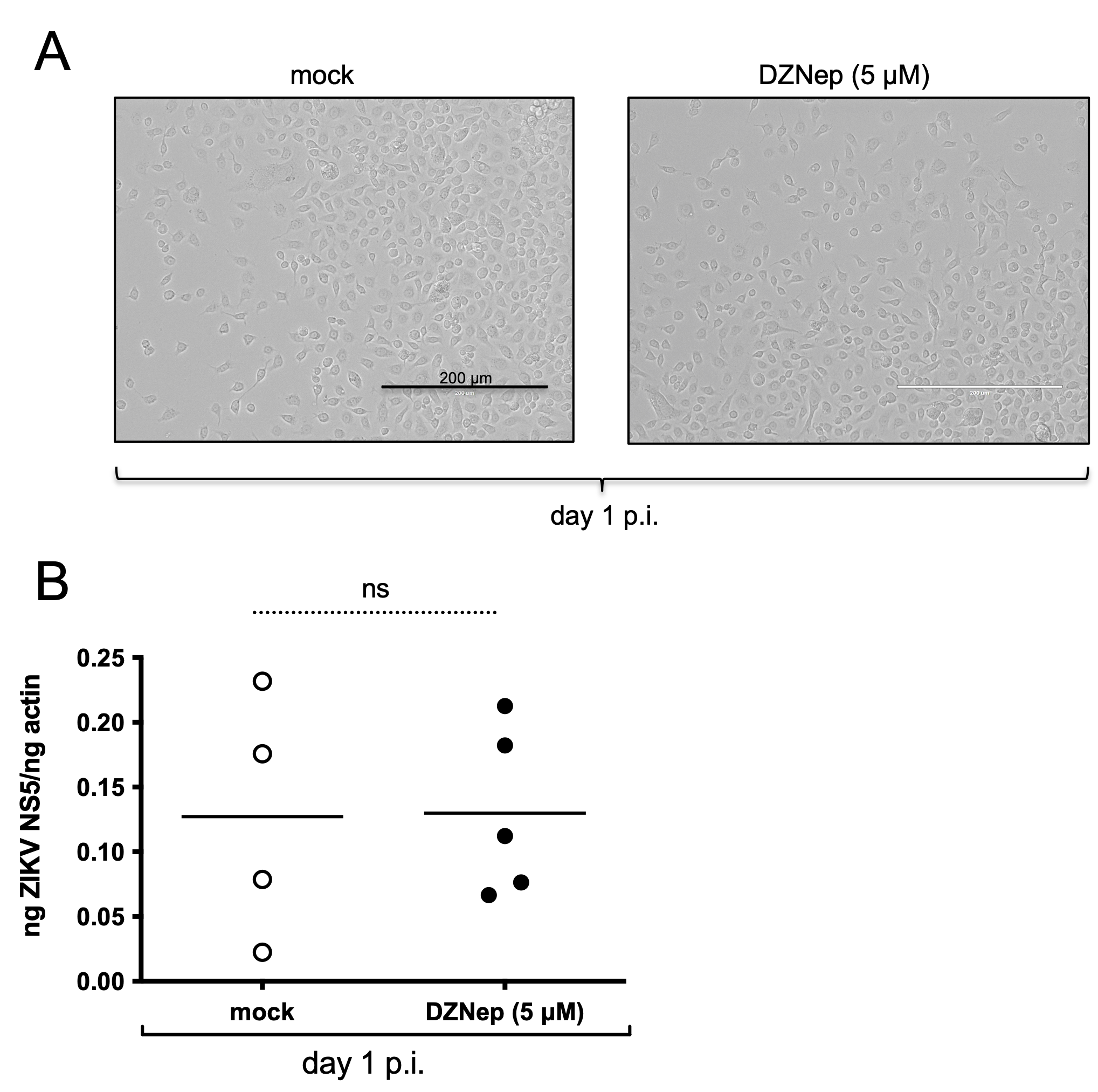

Supplement: S4 Fig — A) Phase contrast microscopic images of ZIKV-infected C6/36 cells treated with either mock or DZNep (5 μM) is shown. Scale bar indicates 200 μm. B) RT-qPCR analysis showing viral loads in mock- (open circles) or DZNep (5 μM, closed circles)-treated ZIKV-infected C6/36 cells at day 1 p.i.. The ZIKV loads were normalized to mosquito actin levels. Horizontal line indicates mean of the values and P value from non-paired Student’s t-test is shown. (TIF) [file pone.0319290.s004.tif]

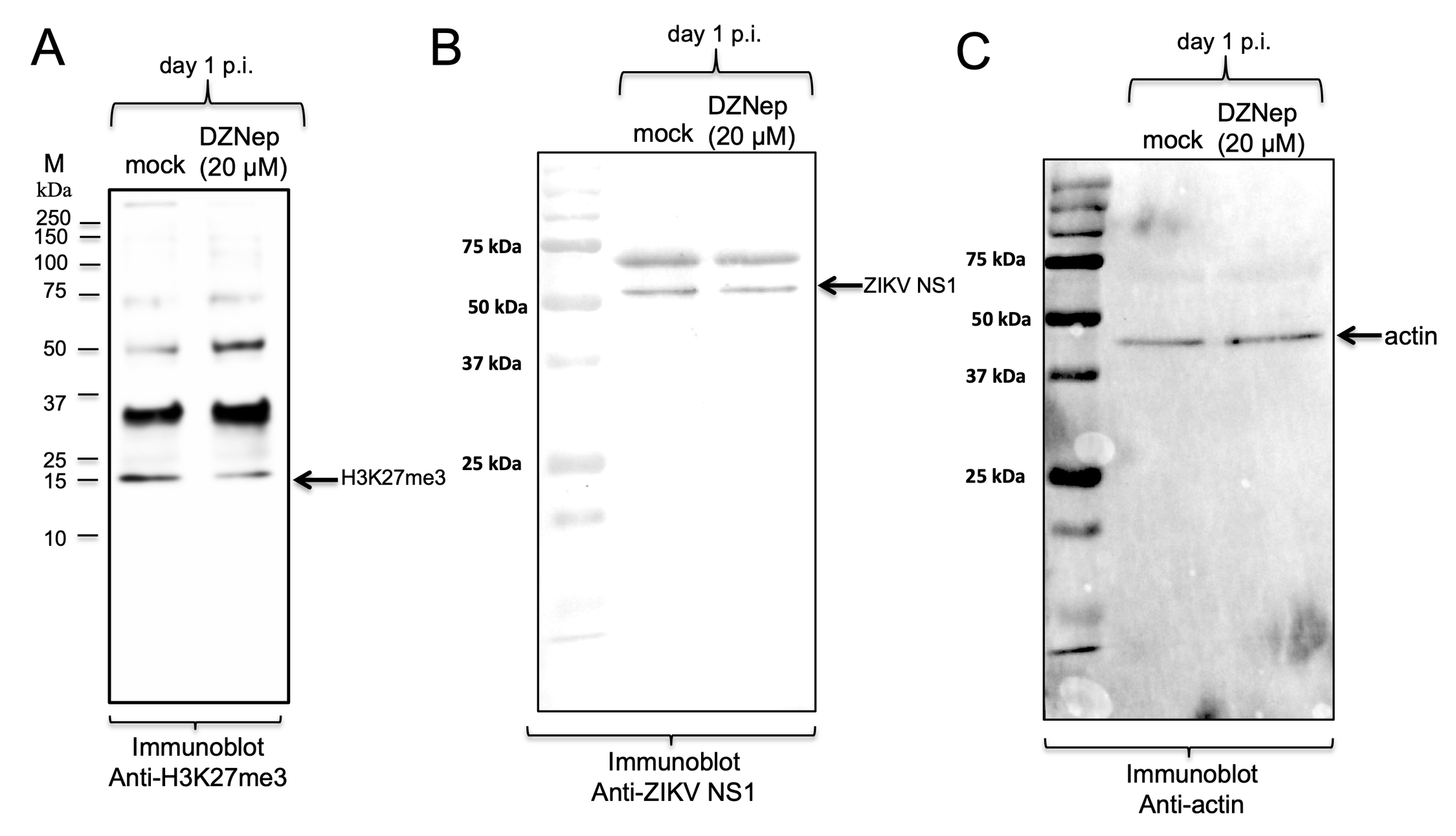

Supplement: S5 Fig — Full-length immunoblot images showing levels of H3K27me3 (A), ZIKV NS1 (B) or actin (C) in mock or DZNep-treated ZIKV-infected cells at days 1 p.i. Arrow indicates the H3K27me3 band at ~15 kDa (A), ZIKV NS1 band around 50 kDa and actin band around 42 kDa (C). The mass of protein marker is indicated in kDa. (TIF) [file pone.0319290.s005.tif]

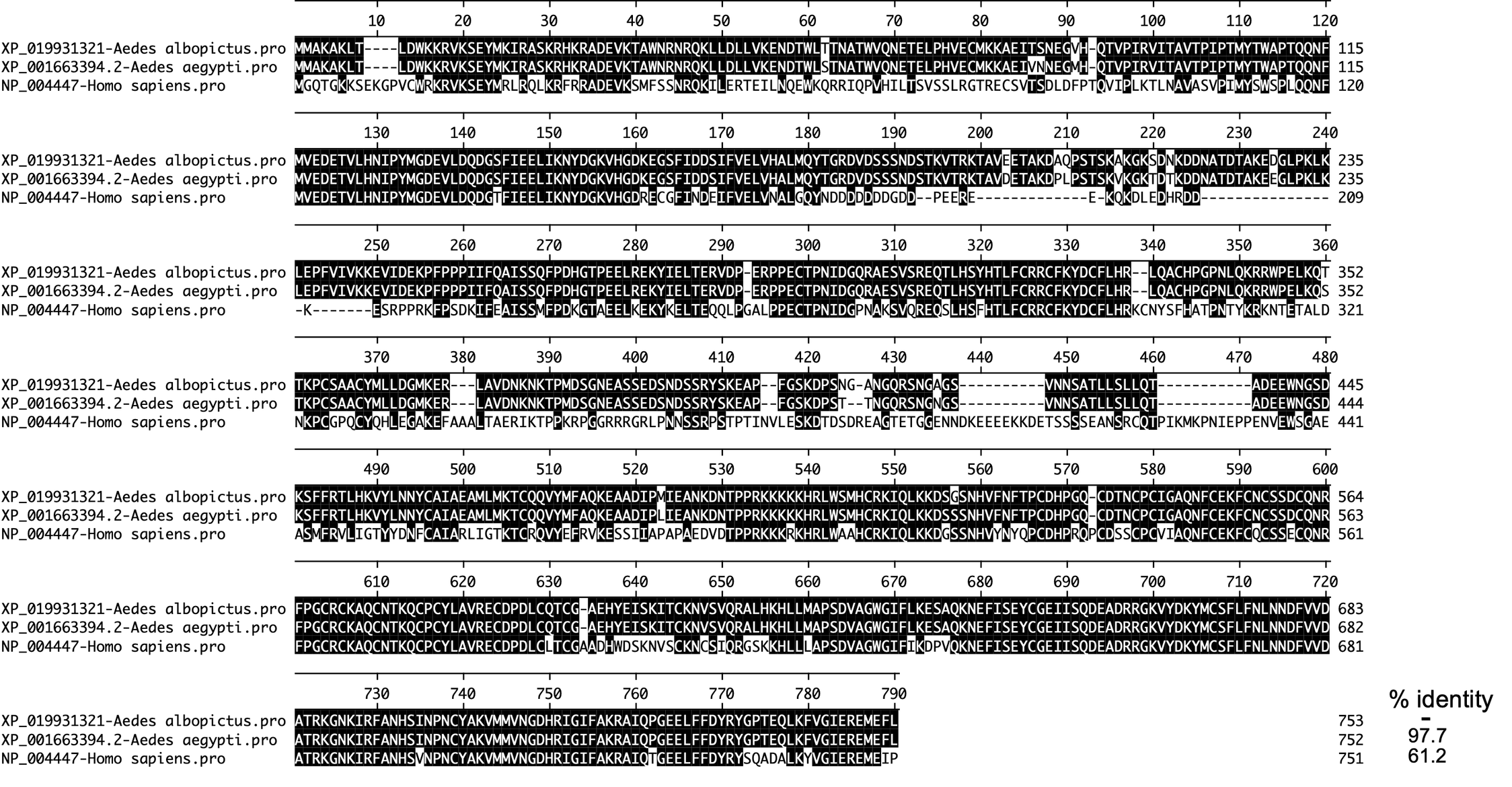

Supplement: S6 Fig — The A. albopictus EZH2 methyl transferase amino acid sequence alignment with A. aegypti and human orthologs using ClustalW program in DNASTAR (Lasergene Genomics Suite) is shown. Residues that match are shaded in black color. GenBank accession numbers for A. albopictus, A. aegypti and H. sapiens sequences are shown. Total length and percent identities of the amino acid sequences are provided at one end of each sequence. (TIF) [file pone.0319290.s006.tif]
