# Supplementary material for: Zika virus modulates arthropod histone methylation for its survival in mosquito cells
Source: PLoS One. 2025 Feb 13;20(2):e0319290. doi: 10.1371/journal.pone.0319290 (PMC11824992; doi:10.1371/journal.pone.0319290)
Supplement: S1 Table — Oligonucleotides used in this study are listed in this table. (PDF) [file pone.0319290.s007.pdf]

Supplementary Table 1. Oligonucleotides used in this study

Sequence (5'-3')	Purpose
CCATGTACGTCGCCATCCA	actin, QPCR
GCGGTGGCCATTTCCTG	actin, QPCR
AARTACACATACCARAACAAAGTGGT	ZIKV NS5, QPCR,
TCCRCTCCCYCTYTGGTCTTG	ZIKV NS5, QPCR,
GGTGAGTTCTGGTGGGCTCGT	SAMe synthase, QPCR
CTTGATGACCTTGTCCATGATGTTCT	SAMe synthase, QPCR
CCCACGCAGCAGAACTTTATG	EZH2 methyl transferase, QPCR
CCTTGGTGCTATCGTTGCTGCT	EZH2 methyl transferase, QPCR
